# Public Health Microbiome Curriculum: Looking Below the Tip of the Iceberg for Approaches to Population Health

**DOI:** 10.1111/1751-7915.70160

**Published:** 2025-06-14

**Authors:** Melissa K. Melby, Swapna Mylabathula, Meghan B. Azad, Sarah Turner, Naama Geva‐Zatorsky, Carolina Tropini, Melissa B. Manus, Martin Blaser, Mark Nichter

**Affiliations:** ^1^ CIFAR Humans & the Microbiome Program Toronto Canada; ^2^ Department of Anthropology University of Delaware Newark Delaware USA; ^3^ Temerty Faculty of Medicine, University of Toronto Toronto Canada; ^4^ Department of Pediatrics and Child Health University of Manitoba Winnipeg Canada; ^5^ Manitoba Interdisciplinary Lactation Centre (MILC), Children's Hospital Research Institute of Manitoba Winnipeg Canada; ^6^ Department of Community Health Sciences University of Manitoba Winnipeg Manitoba Canada; ^7^ Department of Cell Biology and Cancer Science Rappaport Faculty of Medicine, Rappaport Technion Integrated Cancer Center (RTICC), Technion‐Israel Institute of Technology Haifa Israel; ^8^ Department of Microbiology and Immunology University of British Columbia Vancouver Canada; ^9^ School of Biomedical Engineering, University of British Columbia Vancouver Canada; ^10^ Department of Anthropology University of Texas at San Antonio San Antonio Texas USA; ^11^ Departments of Medicine and Pathology & Laboratory Medicine RBHS Robert Wood Johnson Medical School, Rutgers University Rutgers New Jersey USA; ^12^ School of Anthropology, University of Arizona Tucson Arizona USA

**Keywords:** antibiotics, breastfeeding, diet, microbiome, public health

## Abstract

We discuss the opportunity for public health microbiome curricula to bridge the gaps in knowledge that exist between microbiome researchers and the lay public. We propose equipping public health professionals, important facilitators of public outreach and behaviour change, with three public health curriculum modules focused on breastfeeding, antibiotics and diet. These modules shift the focus from microbes as pathogens to potential partners in promoting health across the life course. Current public health messages cover only the ‘tip of the iceberg’ in exploring mechanisms, and this microbiome curriculum dives below the surface to provide fresh perspectives. These microbiome insights allow us to move beyond a focus on microbes as pathogens to understand the numerous collaborative roles played by the microbiome in producing health, and the upstream factors influencing the microbiome, thereby offering mechanistic insights that can be harnessed for public health education.

## Graphical abstract

Current public health messages represent the ‘tip of the iceberg’. We describe three public health microbiome curriculum modules on breastfeeding, antibiotics and diet that examine how upstream factors affect the microbiome and influence health across the life course.

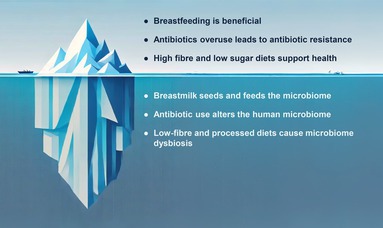

## 1 Introduction

Public health has historically viewed microbes as agents causing infectious disease (pathogens). However, science has recently discovered numerous microbes that live in and on us. Most of these microbes have no known negative effects (termed commensals) and many are beneficial or even essential to human development and health (Gilbert et al. 2018). Together, these microbes constitute the microbiome, or the trillions of microorganisms (and their genomes) that live in and on our bodies. The microbiome is now appreciated as an essential factor in our health.

**FIGURE 1:**
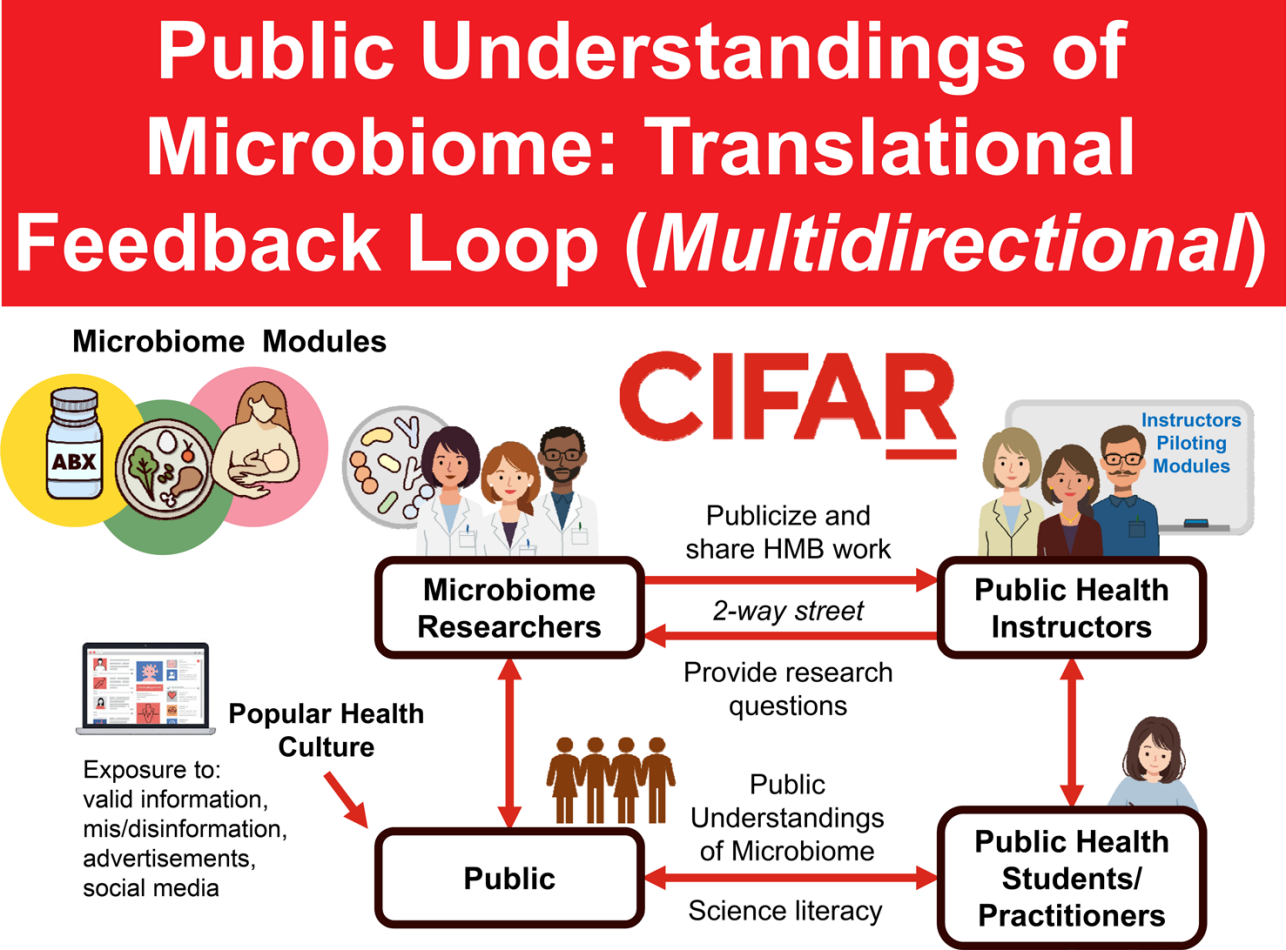
Translational feedback loop for CIFAR Humans & the Microbiome (HMB) Public Health Microbiome modules.

The Canadian Institute for Advanced Research (CIFAR) convenes international, interdisciplinary researchers for five‐year terms to explore important questions facing science and humanity. The current portfolio of 13 research programs includes a program that focuses on *Humans & the Microbiome* (HMB) (CIFAR 2025). HMB is a research program integrating perspectives from microbiology, biomedicine, anthropology and other social sciences, that undertakes interdisciplinary initiatives to explore the complex interactions between human health, disease and the microbiome. HMB includes 20 fellows (from a range career stages), 3–5 advisors (senior scholars) and 3–8 junior scholars (with 2‐year terms), some of whom have become fellows. Meetings are convened twice a year, and this public health microbiome curriculum was an initiative started by an advisor and co‐director, with contributions from three of the younger fellows, including two junior scholars who became fellows. Meetings also include a trainee as recorder, and students and trainees in fellows' research groups are often involved in meetings and catalyst grant projects, as well as this public health microbiome curriculum. For example, one of the co‐authors who worked extensively on the curriculum was a PhD student in one fellow's group and a post‐doc in another's group. By fostering collaboration and knowledge exchange, HMB aims to create societal impact by generating insights that can inform public health strategies, medical treatments and policy decisions. To better understand the current knowledge and interest in the microbiome among public health professionals, we held roundtables that included 23 public health professionals working for Masters of Public Health (MPH) programs, public health agencies, non‐governmental organisations (NGOs), academic institutions and the Council on Education for Public Health (CEPH) accreditation organisation. These sessions allowed us to explore opportunities for how research on the microbiome could inform new approaches to promoting healthy development and aging, as well as broader opportunities for policy and practice. The discussions highlighted a significant gap in current knowledge and understanding about the microbiome despite growing public interest, shaped in part by the marketing of products like probiotics. In response, we convened leading microbiome scientists and public health professionals to develop curriculum modules targeted for MPH students that address critical gaps in public health education on the microbiome (Figure 1).

For students and instructors, the microbiome curriculum provides case studies that emphasise critical thinking with direct relevance for important public health issues. The modules focus on promoting lifelong health starting with a focus on breastfeeding, antibiotic overuse and resistance, and chronic disease prevention and management (e.g., diabetes) through improved diet. These all address ths issue of microbial dysbiosis, or disruption in the composition, diversity, or function of the microbiota that impairs its normal function in relation to host health.

The microbiome provides an opportunity for students of public health to apply systems thinking (Peters 2014), connecting and modelling the influence of biocultural factors such as breastfeeding, antibiotic use, and diet on human health. Understandings of the microbiome's role in public health also have important implications for public health policy and planning, using evidence‐based approaches to recognising potential mechanistic pathways for improving population health.

The curriculum reframes the role of microbes in public health, reframing microbes not solely as pathogens but also as potential ‘partners in health’. It explores how social determinants of health, such as differential access to healthy diets or extended parental leave, become manifested through the microbiome, thereby propagating health inequities. In this way, the modules foster examination of key public health issues and stimulate consideration of ways to address them with a lens of equity, diversity and inclusion.

### 1.1 Aims of the Curriculum

The curriculum aims to promote an understanding of the microbiome as a key mechanism through which differential behaviours and exposures—such as birth mode, breastfeeding, antibiotic use, and diet – shape health outcomes. Understanding these microbiome‐mediated mechanisms can inform more effective interventions for education and policy by:

- Reframing the view of microbes as not just pathogens but as integral to promoting and maintaining health across the life course.
- Highlighting the effects of breastfeeding, antibiotic overuse, and diet on the microbiome.
- Recognising that microbes may play a role in healthy development across the life course, and in both infectious and chronic disease (Finlay 2020).
- Equipping instructors, students and future public health professionals with knowledge to inform public health advocacy and interventions focused on multiple levels from individual behaviours to public policy.

### 1.2 Why Is It Important That Public Health Professionals Know About the Microbiome?

Understanding the role of the microbiome can enhance public health messaging – reinforcing the importance of breastfeeding, antibiotic stewardship, and high‐fibre diets. This message also alerts public health professionals and the public to as‐yet poorly understood threats to individuals and to population health, such as dysbiosis and its consequences. Knowledge about the microbiome can also inform new approaches to interventions, for example expanding beyond the well‐known concern of population‐level antibiotic resistance to consider the chronic disease risks in individuals resulting from antibiotic use.

**FIGURE 2:**
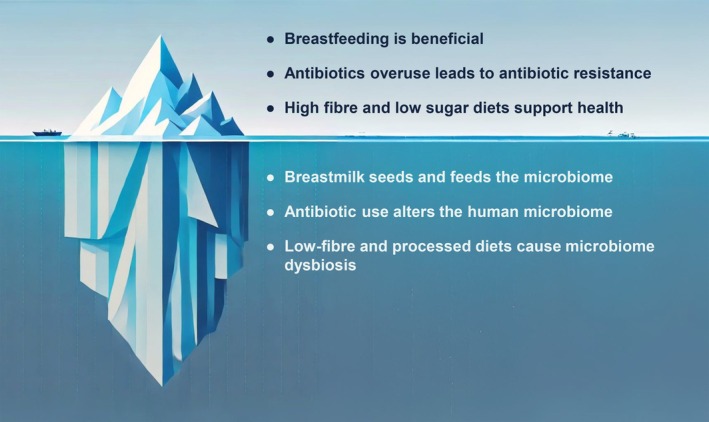
Iceberg analogy showing the ‘tip of the iceberg’ current public health messages (above the surface) about breastfeeding, antibiotics and diet and underlying microbiome roles and impacts (below the surface). (1) Breastmilk seeds and feeds the microbiome, influencing children's long‐term health and development. (2) Antibiotic use alters the human microbiome (eliminating the resident human bacteria (including pathogenic, neutral and beneficial bacteria), at individual and population levels, increasing the risk of infection and chronic disease. (3) Low‐fibre and highly processed diets disrupt the gut microbiome (causing dysbiosis), associated with many chronic diseases.

In Figure 2, commonly understood relationships are shown above the surface, represented by the tip of the iceberg. Insights from microbiome science that are important for public health are under the surface, represented by the iceberg under the water. Interestingly, when an iceberg is formed, it is essentially a chunk of ice that breaks off a glacier – which is called calving. Similarly, our curriculum aims to generate a new perspective or shift in thinking about the microbiome in public health to address the myriad impacts of the microbiome. Just as icebergs are composed of freshwater, and eventually melt into the sea, our microbiome iceberg aims to provide a ‘fresh’ take on public health that can be infused throughout public health curricula. Three topics (breastfeeding, antibiotics and diet ) were chosen for initial modules because of their public health significance across groups, regions and the lifespan. We outline the reasons for these three modules below in more detail.

Breastfeeding provides complete nutrition to young infants along with critical immune protection. Sub‐optimal breastfeeding practices are estimated to cause over 800,000 deaths annually (Victora et al. 2016), primarily in low‐resource settings where non‐breastfed infants may fail to thrive and/or develop fatal infections. Even in high‐resource settings, breastfeeding offers substantive protection against common early‐life infections that strain healthcare systems (e.g., ear infections and respiratory infections which are leading causes of physician visits, antibiotic use and hospitalisations). Longer‐term benefits of breastfeeding include lower risks of chronic diseases such as asthma, obesity and type 2 diabetes, which are among the most prevalent, costly and burdensome diseases facing industrialised societies. While evidence for the health impacts of breastfeeding has accumulated for decades, the underlying mechanisms have remained elusive – but recent research demonstrates a key role for the microbiome, which is both deeply affected by human breast milk and inextricably connected to the programming of immunity and metabolism across the lifecourse (Ames et al. 2023). The public health microbiome breastfeeding module extends knowledge about the benefits of breastfeeding to include understanding of the microbiome mechanisms.

Infectious disease and antibiotic use can impact the composition of the gut microbiome throughout life. According to the World Health Organisation (WHO), infectious diseases caused an estimated 13.7 million deaths globally in 2019, accounting for nearly 24% of all deaths (Ikuta et al. 2022)—many of which could have been prevented with timely antibiotic treatment. Since their introduction in the 1920s, antibiotics have saved over 200 million lives, reducing mortality from bacterial infections by up to 80% in some regions (Laxminarayan et al. 2013). Today, antibiotics are used in every country with global consumption exceeding 70 billion doses annually (Klein et al. 2018). However, 30%–50% of antibiotic use is unnecessary or inappropriate (Blaser et al. 2021), fueling antimicrobial resistance (AMR), which already, by itself, contributes to 1.27 million deaths per year (Murray et al. 2022). Without intervention, AMR could cause at least 10 million annual deaths by 2050 (O'Neill 2016; Tang et al. 2023), surpassing cancer as a leading cause of mortality. Efforts to curb misuse—such as stricter prescribing guidelines and public awareness—are critical to preserving these life‐saving drugs. This is particularly important given our growing understanding of the impact of antibiotics on the microbiome (Blaser 2016). While antibiotic resistance is a well‐known public health crisis, the related public health issue of loss and disruption of the microbiome due to antibiotic overuse is less well‐known, and the public health microbiome antibiotics module fills that gap.

While breastfeeding is one of the earliest and most important nutritional factors influencing the microbiome and human health, after weaning, diet continues to play a major role in shaping long‐term health outcomes. According to the Institute for Health Metrics and Evaluation (IHME), 10.6% of all deaths globally in 2021 were associated with poor diet, with cardiovascular disease being the leading cause of death associated with diet. Conversely, healthy diets have been associated with improved health (IHME 2021). A 2022 study led by IHME researchers found that increasing vegetable consumption from zero to 306–372 g daily was associated with a 23.2% decline in the risk of ischemic stroke, a 22.9% decline in the risk of ischemic heart disease, a 15.9% decline in the risk of hemorrhagic stroke and a 28.5% decline in the risk of oesophageal cancer (Stanaway et al. 2022). While the importance of diet for health is well known to the public health community, the role of the microbiome in mediating the influence of diet on health is not. The public health microbiome diet curriculum module establishes that connection.

### 1.3 Overview of the Modules

**FIGURE 3:**
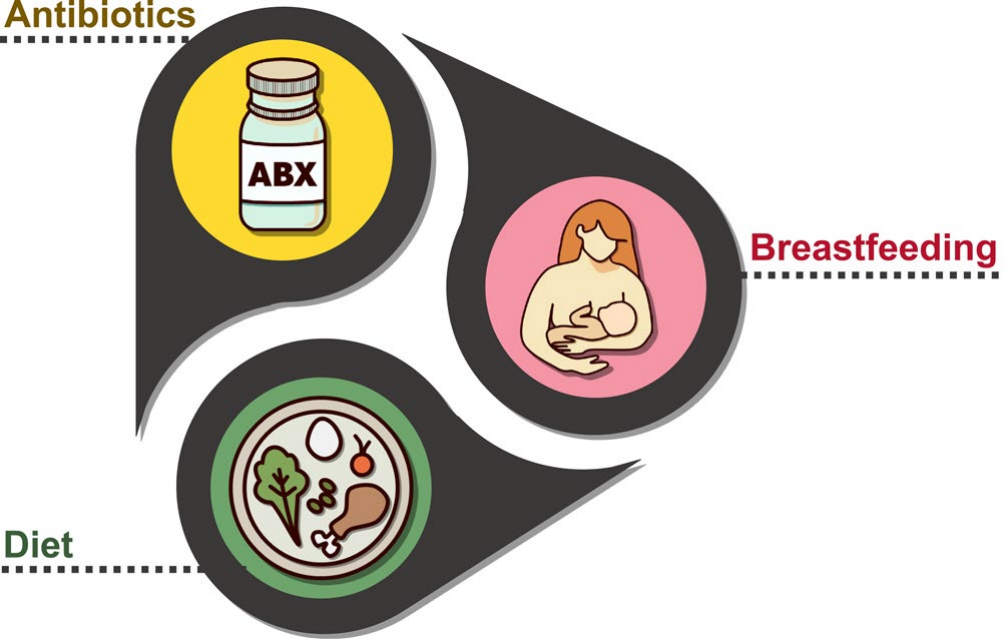
Three CIFAR Humans & the Microbiome (HMB) Program public health microbiome curriculum modules.

The CIFAR HMB program created three modules, focusing on Breastmilk/Breastfeeding, Antibiotics and Diet (Bokulich et al. 2016) to call attention to key messages essential for public health professionals when educating the public. (Figure 3).

Behaviours like antibiotic overuse and dietary changes including less breastfeeding and reduced intake of dietary fibre via consumption of more processed foods have disrupted the human gut microbiome throughout the world. Some microbes are disappearing, and microbiomes are becoming increasingly disrupted and disturbed (inside our guts and in the greater environment). Understanding their roles is critical to identifying the consequences of microbiome damage and how to stop or even reverse this disruption before it is too late and restore the microbial balance that has been the key to human health and success on this planet.

Dysbiotic microbiomes and decreased microbial diversity are often overlooked as risk factors for chronic diseases such as asthma and allergies and obesity and diabetes. Public health professionals can use microbiome research to both recharge existing public health messages by providing mechanistic explanations, and to guide chronic disease prevention and approaches by tailoring advice and policies on breastfeeding, antibiotic use and diet to promote healthy microbiomes.

Since microbes are invisible, it is easy to ignore them—unless something goes wrong. As the lessons learned with the Titanic show us, what lurks below the surface of the water may pose greater threats to survival than what we see above. This curriculum aims to take a deeper dive below the surface of public health and make the invisible visible—by helping students and instructors see how microbes often mediate the social determinants (e.g., access to healthy diets including breastfeeding) that are the focus of much of public health. It also helps public health professionals to understand the mechanisms by which health inequities are produced and to design educational interventions and policies that can prevent, buffer, or reverse these microbiome disruptions.

## 2 Modules

**FIGURE 4:**
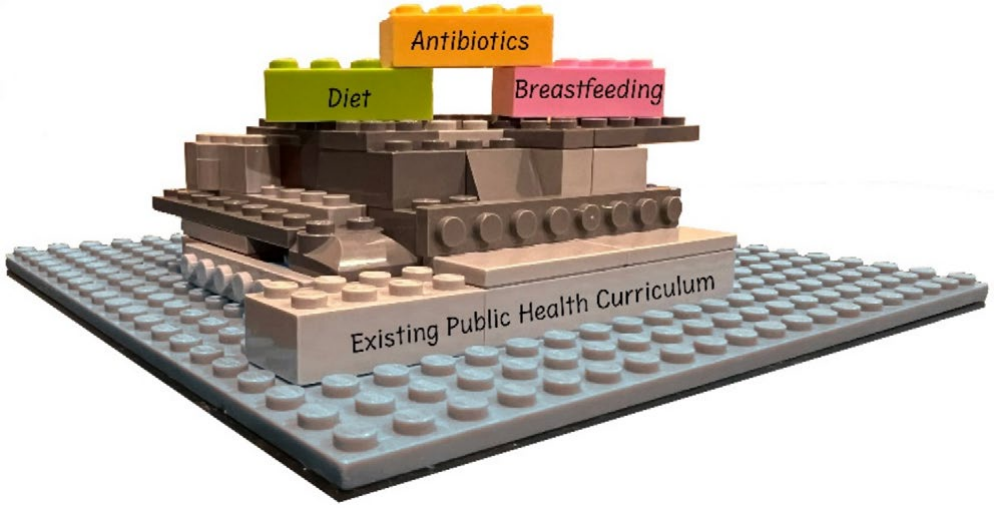
CIFAR HMB public health microbiome modules are designed to be modular, like Lego toys, so that they can be seamlessly incorporated into existing public health curriculum.

The free and publicly available modules (https://cifar.ca/cifarnews/2025/04/10/humans‐the‐microbiome‐educational‐modules‐for‐public‐health‐professionals/) containing modifiable slide decks (50–75 min of lecture time) are inspired by the modular Lego toys (Figure 4) and allow for the introduction of microbiome perspectives into various public health curricula. Instead of a stand‐alone elective course on the microbiome, the breastmilk module can be integrated into and adapted for courses on maternal‐child health or developmental origins of health and disease (DOHaD), the diet module can be incorporated into courses ranging from nutrition to chronic disease, and the antibiotics module can be incorporated into public health history courses, One Health and chronic disease courses.

Core content contains speaker notes for ease of delivery, with optional in‐depth sections for advanced discussion and interactive learning activities. Modules have been piloted in classrooms across North America (e.g., at universities in Arizona, Delaware, Manitoba and Toronto) and beyond (University College Dublin) among undergraduate and graduate students in public health, medical/nutritional anthropology, nursing, as well as medical residents, with positive feedback from students and instructors. Feedback was used to refine and modify the modules. Modules address many Council on Education for Public Health (CEPH) foundational knowledge areas and competencies (CEPH 2025), including developing systems‐level thinking; explaining biological, environmental and behavioural factors affecting population health; and developing evidence‐based approaches to public health.

### 2.1 Breastfeeding and Microbiome Module

Breastmilk is the most impactful factor shaping gut microbiota in early infancy (Ames et al. 2023; Inchingolo et al. 2024; Xu and Wan 2025). Breastfeeding provides complete nutrition—not only for young infants, but also for their microbiomes. In fact, the third most abundant component of human breast milk are compounds that cannot even be digested by human infants; rather, these prebiotic oligosaccharides serve as essential ‘food’ for beneficial microbes residing in the infant gastrointestinal tract (Bode 2015). Absence of such microbes is associated with increased asthma later in childhood (Shenhav et al. 2024; Boulund et al. 2025). Breast milk additionally contains live microbes that may help ‘seed’ the infant microbiome, along with maternal gut and vaginal microbes acquired during childbirth (Stinson et al. 2020). Breast milk composition is dynamic, changing over time to match the evolving needs of the developing infant and its microbiome. Thus, continued breastfeeding throughout infancy and into the second year of life and beyond fosters the gradual development of the infant microbiome which ensures appropriate development of the immune system (Ames et al. 2023). Disruption of this intricate process through early weaning or use of formula (which contains no or few probiotic microbes or prebiotic oligosaccharides) can elevate risk for microbiome‐related immune disorders and chronic diseases in both the short‐ and long‐term (Shenhav et al. 2024). Notably, beyond the microbiome‐supporting qualities of breast milk, the act of breastfeeding involves direct contact that facilitates bidirectional maternal‐child transfer of microbes (Bode et al. 2020; Fehr et al. 2020), highlighting the importance of policies that support actual breastfeeding (e.g., parental leave) and not only the provision of breast milk (e.g., access to breast pumps). While breastfeeding is an individual behaviour, support efforts must focus on creating environments that enable breastfeeding. These include structural level factors such as social mobilisation to change attitudes towards breastfeeding, and setting level factors, such as ensuring that health services and workplaces have policies and practices to support breastfeeding (Rollins et al. 2016).

### 2.2 Antibiotics and Microbiome Module

Antibiotics have saved countless lives and will continue to do so. They are important and crucial for treating diseases caused by bacterial pathogens. However, there are two main deleterious consequences of antibiotics use: (1) antibiotic resistance (Murray et al. 2022) and (2) damage to the resident microbiome (Blaser 2016). Bacteria have short generation times, meaning that they reproduce very frequently. As a result, genetic changes that confer resistance to antibiotics can also evolve at a rapid pace. When antibiotics are administered to kill pathogenic bacteria, some bacteria may contain random mutations that confer resistance to the antibiotics. This is the context in which there selection for resistant bacteria (pathogenic and non‐pathogenic) occurs, as they are the species and strains that survive and reproduce, passing on that portion of their genome that protects them from the effects of antibiotics. These resistant bacteria then increase in prevalence, since the reduction of antibiotic‐sensitive bacteria opens niches for resistant community members to reproduce. This phenomenon of natural selection (or unintended artificial selection) is highly concerning since it contributes to the antibiotic crisis where existing antibiotics will lose their effectiveness against many pathogens (Laxminarayan et al. 2013). Further, many bacteria use horizontal gene transfer to swap portions of their genome with neighbouring bacteria. This creates opportunities for the spread of antibiotic‐resistant genes within bacterial communities, even in the absence of continued selection for this trait. However, antibiotic resistance is only the tip of the iceberg with antibiotic overuse (Haraoui and Blaser 2023). Most antibiotics in widespread use today are broad‐spectrum, meaning they are not directed only to the disease‐causing pathogens, but also broadly affect the resident human microbiome (Blaser 2016)—the microbes that live in and on us and are important in both promoting and maintaining our health. Broad‐spectrum antibiotics cause ‘collateral damage’ of the microbiome, lead to dysbiosis, and consequently, may render people more vulnerable to further infection and contribute to chronic diseases. Antibiotics use is linked to asthma and obesity in children (Aversa et al. 2021) as well as urinary tract infections (Finucane 2017) and Type 2 diabetes (Fenneman et al. 2023) in adults.

### 2.3 Diet and Microbiome Module

Diet plays a crucial role in shaping the gut microbiota, influencing both immediate and long‐term health outcomes. The diet module highlights the important role of dietary fibre in maintaining microbial diversity and preventing dysbiosis—a condition linked to chronic diseases such as diabetes (Crudele et al. 2023), obesity (Van Hul and Cani 2023) and inflammatory bowel disease (Glassner et al. 2020). While traditional public health messaging emphasises the benefits of a high‐fibre diet, microbiome research provides a deeper mechanistic understanding: fibre serves as a primary energy source for beneficial gut bacteria, which, in turn, produce short‐chain fatty acids that regulate metabolism, immune function and gut barrier integrity (Makki et al. 2018). Conversely, highly processed, fibre‐deficient diets contribute to microbiota depletion, increasing susceptibility to inflammation and disease. The module also challenges conventional dietary wisdom by addressing the unintended effects of artificial sweeteners, which, despite being non‐caloric, can alter microbial metabolism and impact glucose homeostasis (Ruiz‐Ojeda et al. 2019). By reframing diet as a key determinant of microbiome composition and function, this module empowers public health professionals to integrate microbiome science into nutritional recommendations, fostering a holistic approach to chronic disease prevention. In doing so, it addresses an educational gap by linking dietary patterns to microbiome‐mediated health outcomes, equipping students and instructors with the knowledge needed to inform evidence‐based dietary policies and interventions.

## 3 Implications and Applications

The HMB Public Health Curriculum has both public health program and policy implications. While there are changes that individuals can make throughout their lives, upstream policy and population‐level interventions and actions are required for equitable access to the environments that promote microbiome health. For example, individual‐level decisions to breastfeed, minimise antibiotic use and consume healthy high‐fibre diets are all important, but systemic changes are needed to support equitable access to these behaviours and exposures. This includes policies to ensure lactation care, provide parental leave, restrict environmental antibiotic use, limit inappropriate antibiotic prescribing and subsidise healthy and culturally appropriate foods. Increased understanding of the important roles the microbiome plays in producing individual and population health is necessary. Such understanding can only come from education (why we have created the public health curriculum) and will inform policies that promote and support breastfeeding, reduced antibiotic overuse, and equitable physical and economic access to healthy food and environments.

While the Titanic's encounter with the iceberg below the surface proved disastrous, even less dramatic encounters with what is lurking below the surface can cause problems. Microbiome dysbiosis may not cause quick death as can a virulent pathogen, but it can contribute to increased health problems, diminished well‐being and shortened healthy life expectancy, as well as a significant burden on healthcare systems. Like a piece of delicate pottery, we might be able to repair our microbiome if it gets broken or disrupted, but it may not be as good as new. It would be better to take care of it proactively. Preventive public health addressing upstream causes involving the microbiome is likely to be more effective than downstream treatments of the resulting problems. Our microbiome functions as a complex and delicate ecosystem—much like a tropical rainforest—and we must feed and support it so it retains the diversity and resilience it needs in the face of inevitable stressors. Ideally, if we take care of our microbiome (by taking care of upstream factors that support it) we might actually be able to ignore it!

This public health microbiome curriculum will inform public health professionals so that they can work to design and implement interventions and policies that can decrease microbiome disruption (e.g., support breastfeeding, improve access to lactation specialists and breast pumps, promote understanding of collateral damage of taking broad spectrum antibiotics ‘just in case’ a sore throat or ear infection might be bacterial, and support high‐fibre and less processed diets, caution consumers about artificial sweeteners). Future potential modules under consideration include microbiome and: Built Environment (Bosch et al. 2024), Non‐Communicable Diseases (Finlay 2020), Aging (Finlay et al. 2019; Melby et al. 2023), Health Inequities and Equity Promotion (Amato et al. 2021), One Health and Indigenous Knowledge and Health, perhaps with a focus on how knowledge about the microbiome interacts with traditional diets.

## 4 Conclusions

This public health microbiome curriculum provides an opportunity to bridge the knowledge gap between scientists and the public and public health professionals, shifting the focus from microbes as pathogens to microbes as essential and beneficial collaborators in maintaining health. Understanding the human microbiome also challenges public health dichotomies between infectious and chronic disease (Finlay 2020).

By encouraging a more ecological approach to microbes and acknowledging the ‘external’ factors that can promote and maintain healthy microbiomes in addition to those that harm the natural microbiome, the curriculum emphasises the importance of maintaining microbial diversity. Microbiome diversity can be maintained by: (i) supporting establishment of an early life microbiome with breastfeeding; (ii) minimising use of broad‐spectrum antibiotics that promote antibiotic resistance and indiscriminately kill neutral and good microbes; (iii) continuing to support it throughout life with diets rich in important food (e.g., prebiotics such as fibre) for the microbiome.

This public health microbiome curriculum encourages public health professionals to dive deeper under the surface of current mainstream public health understanding of human‐microbe relations. It provides fresh perspectives that allow us to move beyond a focus on microbes as pathogens to understand the myriad collaborative roles played by the microbiome in producing health, and the upstream factors influencing the microbiome, thereby offering mechanistic insights that can be harnessed for public health interventions, education and policies.

## Author Contributions

**Melissa K. Melby:** conceptualization, funding acquisition, investigation, writing – original draft, methodology, project administration, supervision, visualization, writing – review and editing, data curation. **Swapna Mylabathula:** conceptualization, investigation, methodology, visualization, writing – review and editing. **Meghan B. Azad:** conceptualization, data curation, investigation, methodology, visualization, writing – original draft, writing – review and editing. **Sarah Turner:** data curation, writing – review and editing. **Naama Geva‐Zatorsky:** conceptualization, data curation, investigation, methodology, visualization, writing – original draft, writing – review and editing. **Carolina Tropini:** conceptualization, data curation, investigation, methodology, visualization, writing – review and editing, writing – original draft. **Melissa B. Manus:** visualization, writing – review and editing. **Martin Blaser:** writing – review and editing. **Mark Nichter:** conceptualization, data curation, investigation, methodology, visualization, writing – review and editing.

## Conflicts of Interest

The authors declare no conflicts of interest.

## Data Availability

The data that support the findings of this study are openly available in CIFAR HMB at https://cifar.ca/cifarnews/2025/04/10/humans‐the‐microbiome‐educational‐modules‐for‐public‐health‐professionals/.
